# An Experiment to Determine the Effect of Extirpating One Ovarium upon the Number of Young Produced

**Published:** 1788

**Authors:** John Hunter


X. *An Experiment to determine the Effect of extirpating one Ovarium upon the Number of Young produced. By* John Hunter, *Esq. F.R.S.* —From the *Philosophical Transactions of the Royal Society of London.* Vol. lxxvii. *for the Year* 1787. Part II. 4to. London, 1787.

IN all animals of distinct sex, the females, those of the bird kind excepted, have, I believe, two ovaria, and of course the oviducts are in pairs.

By

By diftinct fex, I mean when the parts deftined to the purpofes of generation are of two kinds, each kind appropriated to an individual of each fpecies, diftinguifhed by the appellation of male and female, and equally neceffary to the propagation of the animal; the tefticles, with their appendages, conftituting the male; the ovaria, and their appendages, the female fex.

As the ovaria are the organs which, on the part of the female, furnifh what is neceffary towards the production of the third, or young animal; and as females appear to have a limited portion of the middle ftage of life allotted for that purpofe; it becomes a queftion, whether thofe organs are worn out by repeated acts of propagation; or whether there is not a natural and conftitutional period to that power on their part, even if fuch power has never been exerted? If we confider this fubject in every view, taking the human fpecies as an example, we fhall difcover that circumftances, either local or conftitutional, may be capable of extinguifhing in the female the faculty of propagation. Thus we may obferve when a woman begins to breed at an early period, as at fifteen, and has her children faft, that fhe feldom dom breeds longer than the age of thirty or thirty-five; therefore we may fuppofe, either that the parts are then worn out, or that the breeding conftitution is over. If a woman begins later, as at twenty or twenty-five, fhe may continue to breed to the age of forty or more; and there are, now and then, inftances of women, who, not having conceived before, have had children as late in life as at fifty years or upwards. After that, few women breed, even if they fhould not have bred before; therefore, there muft be a natural period to the power of conception. A fimilar ftop to propagation may likewife take place in many other claffes of animals, probably in the female of every clafs, the period varying according to circumftances; but ftill we are not enabled to determine, how far it depends on any particular property of the conftitution, or of the ovarium alone.

As the female of moft claffes of animals has two ovaria, I imagined, that by removing one it might be poffible to determine how far their actions were reciprocally influenced by each other, from the changes which by comparifon might be obferved to take place, either by the breeding period being fhortened, or per-

 haps, haps, in thofe animals whofe nature it is to bring forth more than one at a time, by the number produced at each birth being diminifhed.

There are two views in which this fubject may be confidered. The firft, that the ovarium, when properly employed, may be a body determined and unalterable refpecting the number of young to be produced. In that cafe we can readily imagine, that, when one ovarium is removed, the other may produce its determined number in two different ways; one when the remaining ovarium, not influenced by the lofs of the other, will produce its allotted number, and in the fame time; the other, when it is affected by the lofs, yet the conftitution demands the fame number of young each time of breeding, as if there were ftill two ovaria; confequently it furnifhes double the number it would have been required to fupply, had both been allowed to remain, but muft ceafe from the performance of its function in half the time. The fecond view of the fubject is by fuppofing, that there is not originally any fixed number which the ovarium muft produce; but that the number is increafed or diminifhed according to circumftances; that it is rather the conftitution at large that determines the number; and that, if one ovarium is removed, the other will be called upon by the conſtitution to perform the operations of both; by which means the animal ſhould produce, with one ovarium, the ſame number of young as would have been produced if both had remained.

With an intention to aſcertain thoſe points, as far as I could, I was led to make the following experiment; and for that purpoſe choſe pigs in preference to any other animal, becauſe they are eaſily managed, and breed perfectly well under the confinement neceſſary for experiments. Having ſelected two female ones of the ſame colour and ſize, and likewiſe a boar pig, all of the ſame farrow; after having removed one ovarium only from one of the females, and cut a ſlit in one of its ears, that there might be no miſtake between it and the other, I had them well fed and kept warm, that there might be no impediment to their breeding; and whenever they farrowed, their pigs were taken away exactly at the ſame age.

About the beginning of the year 1779, they both took the boar; but the one which had been ſpayed earlier than the perfect female. The diſtance of time, however, was not great, and they continued breeding at nearly the ſame times. The ſpayed animal continued to breed till September, 1783, when ſhe was ſix years old, which was a ſpace of more than four years. In that time ſhe had eight farrows; but did not take the boar afterwards, and had in all ſeventy-ſix pigs. The perfect one continued breeding till December, 1785, when ſhe was about eight years old, a period of almoſt ſix years, in which time ſhe had thirteen farrows, and had in all one hundred and ſixty-two pigs; after this time ſhe did not breed: I kept her till November, 1786.

I have here annexed a table of the different times of each farrow, with the number of pigs produced.

Spayed Sow.

| Farrows. | Number of young. | Time. |
|---|---|---|
| 1 | 6 | Dec. 1779 |
| 2 | 8 | July 1780 |
| 3 | 6 | Jan. 1781 |
| 4 | 10 | Aug. 1781 |
| 5 | 10 | Mar. 1782 |
| 6 | 9 | Sept. 1782 |
| 7 | 14 | May 1783 |
| 8 | 13 | Sept. 1783 |
| | 76 | |

November

November following ſhe was put to the boar, but brought no pigs. April, 1784, ſhe was again put to the boar, without effect, and never was obſerved to take the boar afterwards, although often with him. November, 1784, ſhe was killed.

Perfect Sow.

| Farrows. | Number of young. | Time. |
|---|---|---|
| 1 | 9 | |
| 2 | 6 | |
| 3 | 8 | |
| 4 | 13 | Dec. 1781 |
| 5 | 10 | June 1782 |
| 6 | 16 | Dec. 1782 |
| 7 | 13 | June 1783 |
| 8 | 12 | Oct. 1783 |
| | 87 | |

Eleven pigs more than were produced by the ſpayed ſow in her eight farrows.

| Farrows. | Number of young. | Time. |
|---|---|---|
| 9 | 12 | Feb. 1784 |
| 10 | 16 | June 1784 |
| 11 | 12 | Dec. 1784 |
| 12 | 16 | May 1785 |
| 13 | 19 | Dec. 1786 |
| | 75 | |

After which ſhe bred no more.

The

| | |
|---|---|
| The firſt eight farrows were - - | 87 |
| The laſt five farrows were - - | 75 |
| Total - - - - - - | 162 |
| The number from the ſpayed one - | 76 |
| More than farrowed by the imperfect animal - - - - | 86 |

It is obſervable, that both ſows rather increaſed in their number each time the older they grew, although not uniformly; the difference between the firſt and laſt in both animals being conſiderable.

From the above table we find, that the ſow with only one ovarium bred till ſhe was ſix years old, from the latter end of 1779 till September, 1783, about four years, and in that time brought forth ſeventy-ſix pigs. The perfect animal bred till ſhe was eight years of age. In the laſt, if conception depended on the ovaria, it was to be expected, that ſhe would bring forth double the number at each birth; or, if ſhe did not, that ſhe would continue breeding for double the time. We indeed find her producing ten more than double the number of the imperfect animal, and continuing to breed much longer.

From

From a circumſtance mentioned in the courſe of this experiment it appears, that the deſire for the male continues after the power of breeding is exhauſted in the female; and therefore does not altogether depend on the powers of the ovaria to propagate, although we muſt at the ſame time allow, that it may be influenced by the exiſtence of ſuch parts.

If theſe obſervations ſhould be conſidered as depending on a ſingle experiment, from which alone it is not juſtifiable to draw concluſions, I have only to add, that the difference in the number of pigs produced by each was greater than can be juſtly imputed to accident, and is a circumſtance certainly in favour of the univerſality of the principle I wiſhed to aſcertain *.

From this experiment it ſeems moſt probable, that the ovaria are from the beginning deſtined to produce a fixed number, beyond which they cannot go, although circumſtances may tend to diminiſh that number; that the conſtitution at large has no power of giving to

* It may be thought by ſome, that I ſhould have repeated this experiment; but an annual expence of twenty pounds for ten years, and the neceſſary attention to make the experiment complete, will be a ſufficient reaſon for my not having done it.

one ovarium the power of propagating equal to two; for, in the prefent experiment, the animal with one ovarium produced ten pigs lefs than half the number brought forth by the pig with both ovaria. But that the conftitution has fo far a power of influencing one ovarium, as to make it produce its number in a lefs time than would probably have been the cafe if both ovaria had been preferved, is evident from the above-recited experiment.

